# Selected feline abstracts from the Companion Animal Genetic Health conference 2018 (CAGH 2018): Irish Veterinary Journal: Edinburgh, Scotland. 14-15 May 2018

**DOI:** 10.1186/s13620-018-0126-0

**Published:** 2018-08-29

## Poster presentations

## P7 Candidate genes for feline hypertrophic cardiomyopathy: analysis of 18 sarcomeric and non-sarcomeric genes

### Jose Matos^1^, Androniki Psifidi^1,2^, David Connolly^1^, Lois Wilkie^1^, Richard Piercy^1^, Virginia Fuentes^1^

#### ^1^Royal Veterinary College, London, UK; ^2^The Roslin Institute, University of Edinburgh, Edinburgh, UK

##### **Correspondence:** Androniki Psifidi

Hypertrophic cardiomyopathy (HCM) is a common heritable myocardial disease in cats. In humans, HCM is typically caused by mutations in cardiac sarcomere protein genes and occasionally in non-sarcomeric genes. In cats only 2 causative mutations for HCM have been identified so far; both in the sarcomeric gene MYBPC3. We hypothesised that HCM in cats is associated with mutations in the same genes as in human HCM. To investigate this, we performed targeted re-sequencing of 18 genes known to cause HCM in humans in a group of 48 cats (25 cases and 23 breed-matched controls) of 8 different breeds. Variant discovery was performed using the GATK tools for best practice. Allelic/genotypic frequencies at each single nucleotide polymorphism (SNP) locus with a likely high, moderate, or modifier functional impact were compared between cases and controls using the Chi-squared and T-tests. Using this re-sequencing data we also performed genome-wide association (GWAS) and selective sweep (Fst) analyses using the PLINK and GEMMA algorithms. A mixed model that accounted for age, weight and breed, and included the genomic relationship matrix among individuals as a random effect was implemented. Significance level was set at P< 0.05 and a Bonferroni correction for multiple testing was performed. More than 9,000 SNPs and insertions and deletions (INDELs) were detected in 15 out of the 18 genes. 10 SNPs located in the TNNT2, TPM1, ACTN, PDLIM3 and CSRP3 genes had a significantly higher frequency in cases compared to controls. The GWAS detected one SNP with a genome-wide significant association with HCM located within the TNNT2 gene and several others with a suggestive significant association. Fst results supported GWAS results with the highest Fst peak also spanning the TNNT2 gene. These results suggest an association of HCM with TNNT2 and other candidate genes across feline breeds. These results should be validated in a larger population.

Keywords: Feline, Genomics and Variation, Inherited Disease

## P8 Single nucleotide polymorphisms in the feline ACP1 gene are associated with diabetes mellitus in lean Domestic Shorthair cats

### Katarina Hazuchova^1^, Ruth Gostelow^1^, Stijn Niessen^1^, David Church^1^, Mike Boursnell^2^, Brian Catchpole^3^, Yaiza Forcada^1^

#### ^1^Department of Clinical Science and Services, The Royal Veterinary College, Hawkshead, UK; ^2^Animal Health Trust, Newmarket, UK; ^3^Department of Pathobiology and Population Sciences, The Royal Veterinary College, Hawkshead, UK

##### **Correspondence:** Katarina Hazuchova

Diabetes mellitus (DM) in cats resembles type 2 DM in humans. A genome-wide association study of DM in Domestic Shorthair (DSH) cats identified 3 single nucleotide polymorphisms (SNPs) located at the end of chromosome A3. The ACP1 gene, coding for a protein tyrosine phosphatase involved in insulin signalling, is in close proximity to these SNPs and was selected for further investigation as a candidate diabetes susceptibility gene.

The coding sequence for the ACP1 gene was identified in the cat genome assembly and Softberry ® software was used for promoter prediction. Gene-specific primers were designed to sequence the 6 exons and putative promoter using Primer-BLAST. Genomic DNA from well-phenotyped diabetic DSH cats (≥3 months on insulin, IGF-1<1000 ng/ml, lean body condition score) and non-diabetic controls (>12 years, normoglycaemic) extracted from EDTA-blood was used for PCR and subsequent Sanger sequencing. Allele frequencies for identified SNPs were compared between diabetics and controls (Chi-square; significance p<0.05).

No SNPs were identified in the coding sequence of 10 diabetics and 10 controls. Six SNPs were identified in the putative promoter (ACP1:c.-227A>C; ACP1:c.-344C>T; ACP1:c.-378G>A; ACP1:c.-420G>C; ACP1:c.-452G>C; ACP1:c.-693T>G). A case-control study (15 diabetics, 30 controls) revealed that the A-allele of c.-227A>C (p=0.00007), G-allele of c.-378G>A (p=0.00001), G-allele of c.-420G>C (p=0.02) and G-allele of c.-452G>C (p=0.0001) were significantly associated with DM. 9/15 diabetics and 6/30 controls were homozygous for the DM-associated haplotype (p=0.007).

Given the interaction of ACP1 with the insulin receptor, the potential functional impact of SNPs within the putative promoter of the ACP1 gene warrants further investigation.

Keywords: Feline, Genomics and Variation, Inherited Disease

